# Real-world burden of adverse events for apalutamide- or enzalutamide-treated non-metastatic castration-resistant prostate cancer patients in the United States

**DOI:** 10.1186/s12885-022-09364-z

**Published:** 2022-03-22

**Authors:** Arif Hussain, Shan Jiang, Della Varghese, Sreevalsa Appukkuttan, Nehemiah Kebede, Kajan Gnanasakthy, Cynthia Macahilig, Reg Waldeck, Shelby Corman

**Affiliations:** 1 University of Maryland Greenebaum Comprehensive Cancer Center, 22 S Greene St, Baltimore, MD 21201 USA; 2 Bayer Healthcare Pharmaceuticals, 100 Bayer Blvd, Whippany, NJ 07981 USA; 3 OPEN Health, 4350 East-West Highway, Suite 1100, Bethesda, MD 20184 USA; 4 RTI Health Solutions, 5 Sylvan Way, Parsippany, NJ 07054 USA

**Keywords:** Non-metastatic castration-resistant prostate cancer, Retrospective study, Chart review study, Adverse events, Enzalutamide, Apalutamide

## Abstract

**Background:**

Second-generation androgen receptor inhibitors (ARIs) have been associated with adverse events (AEs) such as fatigue, falls, fractures, and rash in non-metastatic castration-resistant prostate cancer (nmCRPC) patients as identified in clinical trials. The objectives of this study were to describe the incidence and management of AEs in patients receiving apalutamide and enzalutamide.

**Methods:**

This retrospective chart review study was conducted in nmCRPC-treating sites in the United States. Patients starting apalutamide or enzalutamide between February 1, 2018 and December 31, 2018 were included and any AEs they experienced were recorded. AEs, including those considered to be of special interest as defined in the pivotal clinical trials of the second-generation ARIs, were analyzed and grouped retrospectively in this study. Detailed chart data (patient demographics, clinical characteristics, treatment history, type of AE, outcomes, and resource utilization) were then collected for a randomly selected subset among patients with ≥1 AE to characterize AEs and their management. Descriptive results were summarized.

**Results:**

Forty-three sites participated in the study. A total of 699 patients were included, of whom 525 (75.1%) experienced ≥1 AE. The most common AEs were fatigue/asthenia (34.3%), hot flush (13.9%), and arthralgia (13.6%). In the subset of 250 patients randomly selected from those who experienced ≥1 AE, patients were primarily White (72.0%), the mean age was 71 years, 86.0% had an Eastern Cooperative Oncology Group score of 0–1 at nmCRPC diagnosis, and the average prostate specific antigen (PSA) value at diagnosis was 23.2 ng/mL. PSA-doubling time < 10 months was chosen as reason to initiate treatment in 40% of patients. The median duration of follow-up was 1.1 years, with 14.4% of patients progressing to metastasis by end of study period. Grade 3–4 and Grade 5 AEs occurred in 14.4 and 0.4% of patients, respectively. Actions taken to manage AEs included AE-directed treatment (38.0%), ARI discontinuation (10.4%), dose reduction (7.6%), and AE-related hospitalization (4.8%).

**Conclusions:**

This study highlights the burden of AEs among nmCRPC patients treated with apalutamide or enzalutamide, providing a relevant real-world benchmark as clinical trial evidence and the treatment landcape for nmCRPC continues to evolve.

## Introduction

Prostate cancer (PC) is the most common cancer occurring in men in the United States (US) [1], and is among the leading causes of cancer-related mortality in men [2]. In 2020, there were an estimated 375,304 deaths due to PC globally, with 32,438 in the United States [3]. Non-metastatic castration-resistant PC (nmCRPC) is a distinct clinical state within the PC disease spectrum in men on testosterone suppression therapy (alternatively termed androgen deprivation therapy [ADT]) who develop rising prostate-specific antigen (PSA) in the setting of castration levels of serum testosterone but without evidence of metastatic disease on imaging tests [4]. Patients with nmCRPC are generally asymptomatic but are at risk for subsequent progression to metastatic disease [5].

A primary goal of treating nmCRPC patients is to delay metastatic disease and prolong survival while maintaining or not adversely affecting patients’ quality of life [6]. Prior to 2018, nmCRPC patients were monitored on ADT alone or treated with first-generation anti-androgens in addition to ADT [7–9]. Since 2018, the US Food and Drug Administration (FDA) has approved the second-generation androgen receptor inhibitors (ARIs) apalutamide, enzalutamide, and darolutamide for the treatment of men with nmCRPC. The efficacy and adverse event (AE) profiles of these therapeutic agents have been described in their respective clinical trials [10–12].

Although all the second-generation ARIs target the androgen receptor signaling axis and demonstrate significant anti-PC activity, they can display somewhat different AE profiles. Enzalutamide and apalutamide cross the blood–brain barrier, whereas darolutamide has a lower propensity to do so [13], which may account for some of the differences in central nervous system (CNS)-related AEs (falls and resulting fractures, fatigue, mental impairment) reported to date with the different second-generation ARIs [14]. Avoiding or minimizing AEs becomes especially relevant in treatment selection for relatively asymptomatic disease states; therefore, it is important to understand the real-world consequences of treating nmCRPC patients with the newer ARIs. Decisions about the use of newer drugs should take into account the balance between the potential risks and the demonstrated benefits that allow patients to maintain their quality of life while minimizing significant toxicity and impact on daily activities.

Understanding the burden of AEs associated with second-generation ARIs, including those considered to be of special interest in the pivotal nmCRPC trials, namely hypertension, cardiovascular events, mental impairment disorder, hepatic impairment, neutropenia, seizures/convulsion, fracture, dizziness/vertigo, hypothyroidism, fatigue/asthenia, bone fracture, falls, rash, weight decrease, cerebral ischemia, heart failure, and posterior reversible encephalopathy syndrome [10–12], is important to help clinicians and patients make informed decisions about treatment selection. There are limited real-world studies that have evaluated the incidence of ARI-related AEs and actions taken to address these AEs in patients with nmCRPC. The objectives of this study were to describe the characteristics of nmCRPC patients and their treatment patterns, and to estimate the incidence and management of AEs in patients receiving the second-generation ARIs apalutamide and enzalutamide in a real-world setting. Darolutamide was not included in the study as it was not an approved treatment for nmCRPC in the United States at the time the study was conducted.

## Materials and methods

### Data source

This two-phase, retrospective, multi-site medical chart review study was conducted in the US using data abstracted directly from patient medical records. Patients with physician-diagnosed nmCRPC treated with apalutamide or enzalutamide were enrolled, and any AEs experienced were recorded by physician investigators in patient logs. A randomly selected subset of patients who experienced at least one AE was formed, and detailed chart data were collected for these patients to further describe patient characteristics, AE characteristics, and the actions taken to manage AEs. No patient identifiers were collected.

### Setting and physician eligibility criteria

A geographically dispersed, random sample of medical oncologists and urologists treating nmCRPC were recruited to act as study investigators and were responsible for the recruitment of nmCRPC patients who met the eligibility criteria for inclusion into the study. Physician investigators were required to have managed and/or treated at least five nmCRPC patients, have at least one nmCRPC patient prescribed either apalutamide or enzalutamide, were affiliated with an integrated healthcare system and had access to patients’ complete medical records, attested they could systematically identify and document AEs in the patient chart, and were not currently employed or acting as a consultant or clinical investigator for a pharmaceutical company involved with CRPC.

### Patient eligibility criteria

The study included adult patients (aged ≥18 years at diagnosis) with a physician-confirmed diagnosis of nmCRPC who initiated treatment with apalutamide between February 1, 2018 and December 31, 2018 or with enzalutamide between July 1, 2018 and December 31, 2018 (based on FDA approval dates) with a minimum of 6 months’ follow-up from ARI initiation. Follow-up concluded at the date of last visit, date of death, or the end of the study period, whichever occurred first. Patients were excluded if they had a history of metastasis before CRPC diagnosis, had concomitant or prior history of other primary cancers, or were currently enrolled in an nmCRPC-related clinical trial.

### Data collection and study variables

All AEs experienced by nmCRPC patients treated with apalutamide or enzalutamide were recorded by physician investigators using patient logs, based on documentation of AEs in the patient chart. Adverse events of special interest included in this analysis were grouped as they were in the clinical trials of second-generation ARIs, with categories from the three trials aligned as closely as possible [10–12].

Within the subset of patients who experienced at least one AE, a sample of patients was selected using a random number generator, and detailed data from diagnosis until end of follow-up were collected in these patients. Variables collected included patient demographics, clinical characteristics, ARI treatment history, type and grade of AE (as assessed by the study investigator), actions taken to address AEs, and AE-related healthcare resource utilization (HCRU). PSA level was collected at the time of nmCRPC diagnosis and at the time of ARI initiation. Data were collected post-metastasis among patients whose disease progressed, but AEs occurring in the metastatic setting were excluded from the analysis due to difficulty with confirming attribution of these AEs to anti-androgen treatment use in the non-metastatic setting.

### Statistical analysis

The incidence of AEs was computed among all included nmCRPC patients treated with apalutamide or enzalutamide. Patient demographics, clinical characteristics, AE characteristics and actions taken, and AE-related HCRU were evaluated descriptively among the subset of randomly selected patients with at least one AE.

This study was primarily descriptive in nature. Categorical endpoints were summarized using both the number and percentage in each category. Confidence intervals around the percentage of patients experiencing adverse events were calculated using the Wilson score method. Continuous endpoints were summarized using the mean, standard deviation (SD), and median. Key time-to-event endpoints (e.g., time to AE) were estimated by Kaplan and Meier (KM) methodology and summarized using KM estimates with 95% confidence intervals (CIs). All statistical analyses were performed using SAS version 9.4 (SAS Institute, Inc.; Cary, NC).

### Ethics statement

This study adhered to the principles of the Declaration of Helsinki and was approved by the New England Institutional Review Board in October 2019, with an exemption from informed consent under 45 CFR 46116(f).

## Results

**Fig. 1:**
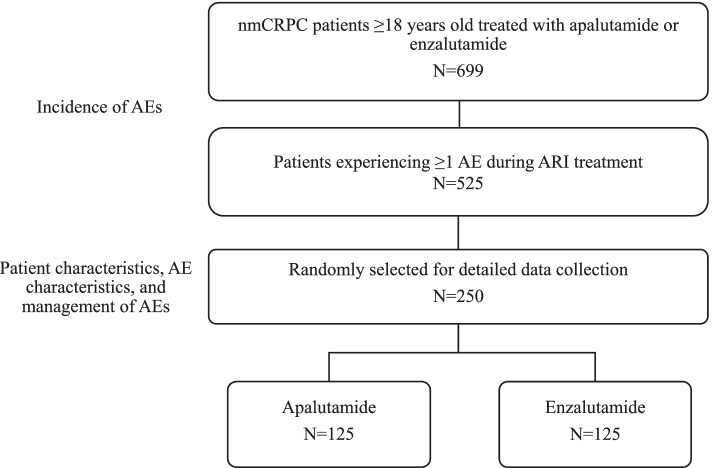
Distribution of included patients. Abbreviations: AE, adverse event; ARI, androgen receptor inhibitor; nmCRPC, non-metastatic castrate-resistant prostate cancer

Forty-three physicians (36 medical oncologists, 6 urologist/urologist-oncologists, 1 radiologist) treating nmCRPC patients were recruited as study investigators. A total of 699 nmCRPC patients who initiated treatment with apalutamide or enzalutamide were enrolled into the study. Of these, 525 patients experienced at least 1 AE, and 250 of these patients were randomly selected for further characterization (Fig. 1).

### Physician characteristics

The majority of the 43 physicians who collected data for the study were male (86.0%) and more than half had been in practice for 15 years or less. Each physician currently managed/treated a median of 74 PC and 26 nmCRPC patients within their practice. All physicians (100%) reported using PSA to monitor their nmCRPC patients, but only 39.5% reported using PSA doubling time (PSA-DT) for routine monitoring of nmCRPC patients.

### Incidence of all-grade AEs in the overall study cohort

The 699 patients included in the study (apalutamide, 368; enzalutamide, 333; both therapies, 2) were followed for a median of 1.1 years (first quartile to third quartile, 0.9 to 1.2 years; for apalutamide, 1.2 years, enzalutamide 1.0 years). Among all 699 patients, 72.0% of men receiving apalutamide and 78.7% of men receiving enzalutamide experienced at least one AE. The most common AEs of any nature were fatigue/asthenia (apalutamide, 30.2%; enzalutamide, 38.7%), flush (apalutamide, 14.1%; enzalutamide, 13.5%), and arthralgia (apalutamide, 14.4%; enzalutamide, 12.9%) (Table 1). Among the AEs of special interest, fatigue/asthenia was the most common, followed by hypertension (apalutamide, 7.3%; enzalutamide, 6.9%) and mental impairment disorders (apalutamide, 5.4%; enzalutamide, 7.5%).

**Table 1:** Proportion of patients receiving ARIs who experienced AEs

	All Patients (***N*** = 699)	Apalutamide^a^ (***N*** = 368)	Enzalutamide^a^ (***N*** = 333)
Any AE, proportion (95% CI)	75.1% (71.8, 78.2%)	72.0% (67.2, 76.4%)	78.7% (74.0, 82.7%)
Adverse events that occurred in ≥5% of patients in either group, proportion (95% CI)			
Hot flush	13.9% (11.5, 16.6%)	14.1% (10.9, 18.1%)	13.5% (10.3, 17.6%)
Arthralgia	13.6% (11.2, 16.3%)	14.4% (11.2, 18.4%)	12.9% (9.7, 16.9%)
Decreased appetite	9.4% (7.5, 11.8%)	6.5% (4.4, 9.5%)	12.9% (9.7, 16.9%)
Diarrhea	6.7% (5.1, 8.8%)	5.4% (3.5, 8.2%)	8.1% (5.6, 11.5%)
Dizziness/vertigo	5.9% (4.4, 7.9%)	5.2% (3.3, 7.9%)	6.6% (4.4, 9.8%)
Peripheral edema	4.0% (2.8, 5.7%)	3.0% (1.7, 5.3%)	5.1% (3.2, 8.0%)
Adverse events of special interest, proportion (95% CI)^b^			
Fatigue/asthenia	34.3% (30.9, 37.9%)	30.2% (25.7, 35.0%)	38.7% (33.7, 44.1%)
Hypertension	7.2% (5.5, 9.3%)	7.3% (5.1, 10.5%)	6.9% (4.6, 10.2%)
Mental impairment disorder^c^	6.4% (4.8, 8.5%)	5.4% (3.5, 8.2%)	7.5% (5.1, 10.8%)
Rash	4.7% (3.4, 6.6%)	6.3% (4.2, 9.2%)	3.0% (1.6, 5.4%)
Cardiovascular events	3.1% (2.1, 4.7%)	2.4% (1.3, 4.6%)	3.9% (2.3, 6.6%)
Headache	3.1% (2.1, 4.7%)	3.5% (2.1, 5.9%)	2.7% (1.4, 5.1%)
Falls	2.3% (1.4, 3.7%)	1.6% (0.7, 3.5%)	3.0% (1.6, 5.4%)
Fracture	1.1% (0.6, 2.2%)	1.9% (0.9, 3.9%)	0.3% (0.1, 1.7%)
Weight decrease	1.6% (0.9, 2.8%)	1.4% (0.6, 3.1%)	1.8% (0.8, 3.9%)
Hypothyroidism	1.1% (0.6, 2.2%)	1.4% (0.6, 3.1%)	0.9% (0.3, 2.6%)
Seizure/convulsion	0.7% (0.3, 1.7%)	0.3% (0.0, 1.5%)	1.2% (0.5, 3.0%)
Hepatic impairment	0.4% (0.1, 1.3%)	0.0% (0.0, 1.0%)	0.9% (0.3, 2.6%)

*Abbreviations: AE* Adverse event; *ARI* Androgen receptor inhibitor; *CI* Confidence interval

^a^Two patients received both apalutamide and enzalutamide. The specific AEs have been attributed to the respective therapy cohort, and therefore the Ns add to > 100%

^b^No patients experienced neutropenia, cerebral ischemia, heart failure, or posterior reversible encephalopathy syndrome

^c^Included cognitive and attention disorders, memory impairment, mental and cognitive changes, and mental impairment disorder

### Patient and treatment characteristics in the randomly selected subset population

In the subset of randomly selected 250 patients with at least one AE, 125 patients received apalutamide and 125 received enzalutamide. On average, these patients were 71 years old, and nearly three-fourths were White/Caucasian (72.0%) and covered by Medicare (74.4%) (Table 2). At the time of nmCRPC diagnosis, 86.0% of patients had an Eastern Cooperative Oncology Group (ECOG) score of 0–1, a majority had a Gleason score of 8–10, and mean PSA values were 23.21 ng/mL. Only 41 (16.4%) patients had at least two PSA values (at nmCRPC diagnosis and at ARI initiation) that could be used to calculate PSA-DT; 6 (2.4%) patients had a negative PSA-DT and 10 (4.0%) had a PSA-DT greater than 10 months. Patients were followed up for a median of 13 months from initiation of ARI.

**Table 2:** Patient demographic and clinical characteristics in the 250-patient subset

	All Patients (***N*** = 250)	Apalutamide (***N*** = 125)	Enzalutamide (***N*** = 125)
Age at most recent visit, years			
Mean (SD)	70.84 (7.84)	70.41 (8.21)	71.28 (7.45)
Median (Q1-Q3)	70.0 (66.0–76.0)	70.0 (66.0–76.0)	71.0 (66.0–77.0)
Race, N (%)			
White or Caucasian	180 (72.0)	91 (72.8)	89 (71.2)
Black or African/Caribbean origin	65 (26.0)	31 (24.8)	34 (27.2)
Asian	2 (0.8)	0 (0.0)	2 (1.6)
American Indian/Alaska Native	1 (0.4)	1 (0.8)	0 (0.0)
Unknown	2 (0.8)	2 (1.6)	0 (0.0)
Healthcare coverage at most recent visit, N (%)			
Medicaid	28 (11.2)	15 (12.0)	13 (10.4)
Medicare	186 (74.4)	92 (73.6)	94 (75.2)
Medigap	5 (2.0)	3 (2.4)	2 (1.6)
Private	24 (9.6)	10 (8.0)	14 (11.2)
Traditional fee-for-service	5 (2.0)	3 (2.4)	2 (1.6)
Health maintenance organization	9 (3.6)	2 (1.6)	7 (5.6)
Preferred provider organization	27 (10.8)	18 (14.4)	9 (7.2)
Veterans Affairs	2 (0.8)	0 (0.0)	2 (1.6)
Body mass index at most recent visit			
Mean (SD)	27.47 (3.90)	27.46 (3.76)	27.47 (4.05)
Median (Q1-Q3)	26.8 (25.0–28.9)	27.0 (25.3–28.8)	26.8 (24.8–29.0)
Charlson Comorbidity Index, N (%)^a^			
0	162 (64.8)	85 (68.0)	77 (61.6)
1	60 (24.0)	27 (21.6)	33 (26.4)
2+	28 (11.2)	13 (10.4)	15 (12.0)
ECOG score at nmCRPC diagnosis, N (%)			
0	83 (33.2)	34 (27.2)	49 (39.2)
1	132 (52.8)	71 (56.8)	61 (48.8)
2	25 (10.0)	11 (8.8)	14 (11.2)
3+	2 (0.8)	2 (1.6)	0 (0.0)
Unknown	8 (3.2)	7 (5.6)	1 (0.8)
Gleason score at nmCRPC diagnosis, N (%)			
2 to 6	28 (11.2)	14 (11.2)	14 (11.2)
7	87 (34.8)	44 (35.2)	43 (34.4)
8 to 10	94 (37.6)	47 (37.6)	47 (37.6)
Unknown	41 (16.4)	20 (16.0)	21 (16.8)
PSA at nmCRPC diagnosis (ng/mL)			
N	241	118	123
Mean (SD)	23.21 (44.15)	20.99 (24.91)	25.35 (56.84)
Median (Q1-Q3)	12.0 (6.7–26.0)	13.0 (7.0–28.0)	11.0 (6.5–22.4)

*Abbreviations: ECOG* Eastern Cooperative Oncology Group; *Q1-Q3*, Range between the first quartile (Q1) and third quartile (Q3); *nmCRPC* non-metastatic castrate-resistant prostate cancer; *PSA* Prostate specific antigen; *SD*, Standard deviation

^a^Charlson Comorbidity Index was calculated using patient comorbidities present from nmCRPC diagnosis through the end of the study period

Nearly all patients in the subset (95.6%) were prescribed apalutamide or enzalutamide as their first line of therapy in the nmCRPC setting. The most common physician-reported rationales for initiating ARI treatment were to prevent/delay metastasis (overall, 63.2%; apalutamide, 66.4%; enzalutamide, 60.0%) and for a PSA-DT less than 10 months (overall, 40.4%; apalutamide, 40.8%; enzalutamide, 40.0%). Median duration of ARI therapy was 13.0 months overall (Q1-Q3, 10.6–15.5 months), 13.6 (10.8–17.3) months for apalutamide, and 12.8 (10.3–14.1) months for enzalutamide. Overall, 14.4% of patients progressed to metastasis by end of study (apalutamide, 16.0%; enzalutamide, 12.8%).

### AE characteristics and actions taken to address AEs and associated HCRU in the subset population

A total of 444 AEs were reported in the subset of 250 patients who experienced at least one AE. Nearly 12% of patients treated with ARIs had at least one physician-defined serious AE, and 8.1% of the 444 AEs were judged by the physician to be serious. Similar to the full patient population, the most common AEs of any nature in the subset population were fatigue/asthenia (50.8% of patients), flush (20.8%), and arthralgia (18.8%). Grade 3–4 and grade 5 AEs occurred in 36 (14.4%) and 1 (0.4%) patients, respectively. The median time from ARI initiation to first AE was 56 days (95% CI, 49–70 days).

**Fig. 2:**
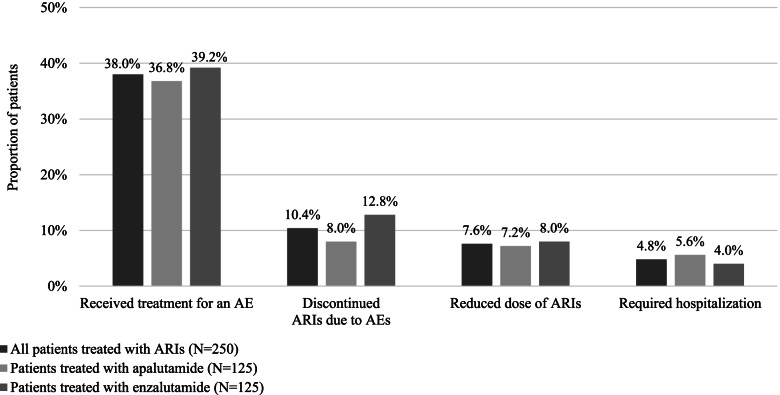
Actions taken to address AEs occurring during treatment with ARI among subset of patients with ≥1 AE^a^. Abbreviations: AE, adverse event; ARI, androgen receptor inhibitor. ^a^ Actions taken to address AEs are not mutually exclusive; multiple actions could have been taken

In the 250-patient subset, 95 (38.0%) patients required treatment for AEs (Fig. 2). Actions taken to address AEs included hospitalizations (4.8% of patients), discontinuation due to AEs (10.4%), and dose reduction (7.6%). Of the 444 AEs reported, 32 (7.2%) required discontinuation of therapy, 26 (5.9%) required dose changes, and 116 (26.1%) required treatment. Among the 116 AEs requiring treatments the most frequent were hypertension (23 AEs, 19.8%), arthralgia (14 AEs, 12.1%), diarrhea (9 AEs, 7.8%), and peripheral edema (9 AEs, 7.8%); the most common AE requiring discontinuation was rash (4 of 32 AEs; i.e., 12.5%). More than half of the 444 AEs resolved without sequelae (51.6%), while 41.4% did not resolve.

**Fig. 3:**
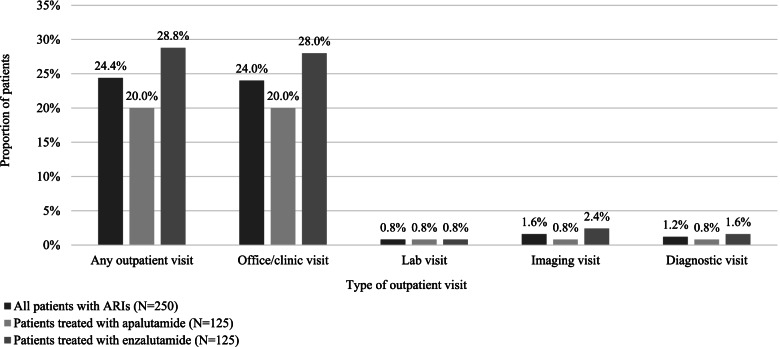
Outpatient resource use for AE management among subset of patients with ≥1 AE. Abbreviations: AE, adverse event; ARI, androgen receptor inhibitor

Among the 12 (4.8%) patients who required hospitalization for their AE, the mean length of hospital stay was 4.58 days (SD, 2.35 days). AEs requiring hospitalization included seizure, fracture, falls, hypertension, and other cardiovascular events. Approximately one-quarter of the patients (24.4%) had at least one outpatient visit associated with AE management, which comprised of office/clinic visits, lab visits, imaging visits, or diagnostic visits (Fig. 3).

### Reasons for ARI discontinuation in subset population

**Fig. 4:**
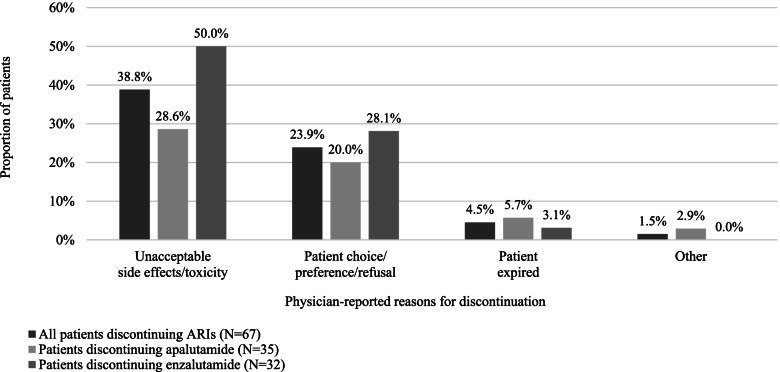
Physician-reported non-progression-related reason for ARI discontinuation among patients who discontinued^a^. Abbreviation: ARI, androgen receptor inhibitor. ^a^ Reasons for discontinuation are not mutually exclusive; multiple reasons could have been given

More than one-quarter (26.8%) of the 250 patients discontinued ARIs for any reason (apalutamide, 28.0%; enzalutamide, 25.6%). The most common reason for treatment discontinuation was disease progression (36 patients, 53.7%). Among the patients discontinuing for other reasons, the most common reason was AEs (38.8%; Fig. 4); other reasons included patient choice and patient death.

## Discussion

This is the first real-world study to examine the incidence and burden of AEs among a nmCRPC population treated with next generation ARIs using data abstracted directly from patient medical charts. Overall, the results show that nmCRPC patients treated with apalutamide and enzalutamide have high risk of developing AEs, with nearly 40% requiring treatment, 10% discontinuing the ARI treatment altogether, and 5% needing hospitalization. Twelve patients required hospitalization for their AEs, of whom 2 experienced seizures and 3 experienced falls/fractures that led to their hospitalization. Amongst the ARI-treated group, 14% also experienced disease progression during the study follow-up period. These findings provide a benchmark for the range and frequencies of AEs that can occur among the ARI-treated nmCRPC patient population in the real-world setting.

The most prevalent AE in this study population was fatigue, similar to what was observed in other real-world studies and clinical trials. The AE rates reported in the current retrospective study are lower than what has been reported in the pivotal clinical trials for apalutamide and enzalutamide [10, 11]. Results of the SPARTAN trial showed that 96.5% of nmCRPC patients treated with apalutamide had at least one AE, with fatigue (30.4%), hypertension (24.8%), and rash (23.8%) reported as the most common AEs [11]. The PROSPER trial conducted to evaluate the effectiveness of enzalutamide among nmCRPC patients showed that 87.0% had at least one AE, with fatigue (33.0%), hot flush (13.0%), and nausea (11.0%) reported as the most common AEs [10]. A previous real-world study by Pilon et al. conducted using insurance claims data defined a subset of CNS-related AEs to include amnesia or memory impairment, anxiety, ataxia, cognitive disorders, confusion, convulsions, disturbance in attention, dizziness, falls, fatigue/asthenia, hallucinations, headaches, insomnia, pain, paresthesia, seizures, weakness, or other CNS disorders [15]. Using this broad definition, the investigators found that, among patients with at least 3 months of exposure to abiraterone, enzalutamide, or bicalutamide, 11.7, 16.6, and 10.1% experienced at least one CNS-related AE. The percentage of patients experiencing fatigue/asthenia, dizziness, headache, and falls were lower in that study compared to this one. In addition, another real-world study of patients with PC (of whom 89.7% received bicalutamide) found fatigue/asthenia (15.6%), rashes (10.9%), insomnia (9.8%), fracture (8.3%), pain (6.6%), and weakness (5.8%) to be the most common AEs [16]. The differences between the specific AE rates reported in this real-world study and those observed in clinical trials could be attributed, in part, to the differences in study design (i.e., in randomized clinical trials, close prospective follow-up of patients typically captures more events than in a real-world setting) as well as inherent differences in the patient populations in clinical trials versus real-world studies, which may cause further discrepancy. Lastly, and importantly, the lower rates of AEs reported in the present study could also be explained by the shorter median duration of follow-up in this study (13 months) compared with the pivotal trials of enzalutamide (18.5 months) [10] and apalutamide (20.3 months) [11].

At the time this study was initiated, darolutamide was not yet approved for use in the US and thus darolutamide-treated patients were not included in the study. While apalutamide, enzalutamide, and darolutamide have not been compared head-to-head in a clinical trial, an indirect treatment comparison across the pivotal studies of enzalutamide, apalutamide and darolutamide was performed. Within the confines of this type of analysis, falls, fractures and rash rates were statistically significantly lower in favor of darolutamide vs apalutamide. Fall, dizziness, mental impairment, fatigue and severe fatigue rates were statistically significantly lower in favor of darolutamide vs enzalutamide [14]. This study adds to the collective body of knowledge by examining the comparative safety of emerging nmCRPC therapies in a real-world setting.

Since the goal of nmCRPC patient management is to prolong metastasis and overall survival while maintaining quality of life, patients will benefit from therapies that have a safety profile that does not interfere with their daily activities. This study highlights the challenge in the treatment of prostate cancer where effective therapies are becoming available but further enhanced safety profiles remains an unmet need and important goal. Future real-world studies may further investigate the respective frequencies of these AEs of interest in nmCRPC among the three second-generation ARIs.

In addition to the AE rates, this study provides insight into real-world trends and practices. Less than 40% of the physician investigators reported using PSA-DT as part of their routine management procedures. The pivotal trials for apalutamide and enzalutamide in nmCRPC included patients at high risk of metastasis, defined as a PSA-DT of 10 months or less [10, 11]. As such, the results of this study suggest that in clinical practice, physicians are prescribing ARIs more broadly in patients they feel to be at high risk of metastasis.

This study has several strengths. First, this is the first real-world study in the US to examine the incidence and burden of AEs among a nmCRPC population treated with ARIs using data abstracted directly from patient charts. The chart review approach allows for the collection of rich clinical data and attribution of AEs to treatment and HCRU to AEs by treating physicians, which cannot be achieved using secondary data such as claims and electronic health records. Unlike in clinical trials, the data for this study were not collected for the purpose of research and therefore are reflective of real-world patterns. Further, we aligned our definition of AEs of special interest with the pivotal clinical trials of second-generation ARIs to allow for comparisons across studies [10–12]. Since this is a chart review study, we were able to collect detailed clinical information including treatments, severity and grade of AEs, and ECOG and Gleason scores from patient charts that may not be commonly available in other sources (e.g., claims data). In this study, we were able to include physician-diagnosed nmCRPC patients without relying on coding in administrative claims, and included patients newly initiating ARIs for the on-label treatment of nmCRPC. Apalutamide and enzalutamide received their FDA approvals for treatment of nmCRPC fairly recently (2018) and this is one of the first studies to report results for these drugs since their approval. Finally, the results of the study are potentially generalizable to nmCRPC patients who received treatment with apalutamide or enzalutamide in the US, as the study population is representative of the nmCRPC population in the US with respect to age, ECOG status, body mass index, and PSA at diagnosis of nmCRPC. As such, this study adds to a building body of evidence on the safety of second-generation ARIs.

This study has some limitations that should be acknowledged in interpreting its results. First, AEs are difficult to identify retrospectively and are likely to be under-documented in studies such as chart reviews, electronic medical records analyses, and claims database analyses. For instance, AEs such as the incidence of rash noted in the current study among patients receiving apalutamide (6.0%) is considerably lower than in the PROSPER trial (23.8%); this in part could be due to minor rashes being potentially overlooked and/or undocumented in the medical record. On the other hand, the presence of certain comorbidities such as hypertension could also have influenced investigators’ attribution of such AEs to ARIs, when in fact they may have been pre-existing. Second, the treatment patterns, AEs, and associated HCRU in nmCRPC patients represent the practices of participating physicians/sites and may vary from non-participating sites. Further, we collected PSA values only at nmCRPC diagnosis and at ARI initiation, and thus had limited data to calculate PSA-DT. For some outcomes, such as reason for discontinuation among patients who stopped treatment, the sample size was small and thus proportions should be interpreted with caution. Moreover, given not only the small sample sizes but also the duration of follow-up in the study being slightly longer in the apalutamide group compared to the enzalutamide group, this study should not be used to compare the relative safety profile of the two ARIs.

## Conclusions

The present analysis provides data on the incidence and impact of AEs in US patients with nmCRPC receiving apalutamide and enzalutamide and highlights the practice patterns of physicians managing such patients, particularly in the context of the evolving treatments in this disease space. Patients treated with apalutamide and enzalutamide were shown to be at high risk of AEs, with nearly 40% requiring treatment, 10% discontinuing the ARI treatment altogether, and 5% needing hospitalization. The data provide a relevant benchmark that will allow further assessment of additional therapies targeting the androgen receptor axis that are becoming increasingly available for patients with nmCRPC.

## Data Availability

The datasets used and/or analysed during the current study are available from the corresponding author upon reasonable request.
